# Supplementary material for: Real-Time Tracking of Selective Auditory Attention From M/EEG: A Bayesian Filtering Approach
Source: Front Neurosci. 2018 May 1;12:262. doi: 10.3389/fnins.2018.00262 (PMC5938416; doi:10.3389/fnins.2018.00262)
Supplement: Supplementary file 1 [file Presentation_1.pdf]

Supplementary Material: Real-Time Tracking of Selective Auditory Attention from M/EEG: A Bayesian Filtering Approach

Sina Miran, Sahar Akram, Alireza Sheikhattar, Jonathan Z. Simon, Tao Zhang, and Behtash Babadi*

*Correspondence:
Author Name: Behtash Babadi
behtash@umd.edu

This supplementary document contains the derivations of our proposed estimation framework as well as additional simulation studies. In Section 1, we present the parameter estimation procedures used for the encoding and decoding models. Section 2 includes the inference algorithms for state estimation using fixed-lag smoothing, and Section 3 discusses the smoothing effect of the proposed state-space model. Finally, we apply our proposed techniques to simulated MEG data in Section 4.

1 DYNAMIC ENCODING AND DECODING MODELS: PARAMETER ESTIMATION

Recall that the encoder/decoder estimation problems can be posed as the following optimization problem:

$$\hat{\boldsymbol{\theta}}_k = \arg\min_{\boldsymbol{\theta}} \sum_{j=1}^{k} \lambda^{k-j} \| \mathbf{y}_j - \mathbf{X}_j \boldsymbol{\theta} \|_2^2 + \gamma \| \boldsymbol{\theta} \|_1 , \quad k = 1, 2, \ldots, K. \tag{S1}$$

At each window k, for $k = 1, \ldots, K$, the encoding/decoding coefficients $\hat{\boldsymbol{\theta}}_k$ are updated based on the new measurements, i.e., $\mathbf{y}_k$ and $\mathbf{X}_k$, and previous measurements through the forgetting factor mechanism while applying sparsity-promoting priors on the coefficients.

There are several standard optimization techniques that can be used to find the minimizer in (S1). Off-line algorithms such as interior point methods do not meet the real-time requirements of our dynamic estimation. The SPARLS algorithm has been introduced in (Babadi et al., 2010) to solve the problem in (S1) through EM iterations, and it has been successfully adopted in (Akram et al., 2017) to estimate encoding coefficients in a dynamic fashion. However, the EM algorithm and the constant step-size in SPARLS may result in low convergence rates. Hence, to adapt our estimation procedure for real-time applications, we use the Forward-Backward Splitting (FBS) method (Combettes and Pesquet, 2011), also known as the proximal gradient method, to solve for $\hat{\boldsymbol{\theta}}_k$ in (S1). FBS is suited for optimization problems where the objective function can be expressed as the sum of a differentiable term, e.g., the log-likelihood term in (S1), and a simple non-differentiable term, e.g., the ℓ_1-norm in (S1). This type of problems frequently arise in signal processing and machine learning (Jenatton et al., 2010; Duchi and Singer, 2009; Figueiredo et al., 2007).

In summary, each FBS iteration for the optimization problem in (S1) includes two steps: 1) taking a descent step along the gradient of the log-likelihood term, and 2) applying a soft-thresholding shrinkage operator (Goldstein et al., 2014; Sheikhattar et al., 2015). This procedure provides an algorithm that uses recursive and low-complexity updates in an online fashion to solve Eq. (S1) upon the arrival of a new data window. The optimization problem in (S1) can be rewritten as:

$$\hat{\boldsymbol{\theta}}_k = \arg\min_{\boldsymbol{\theta}} \; \boldsymbol{\theta}^T \mathbf{A}_k \boldsymbol{\theta} + \mathbf{b}_k^T \boldsymbol{\theta} + \gamma \parallel \boldsymbol{\theta} \parallel_1, \quad k = 1, 2, \ldots, K, \tag{S2}$$

where $\mathbf{A}_k$ and $\mathbf{b}_k$ can be updated recursively. Algorithm 1 summarizes the steps of the FBS algorithm to solve for $\boldsymbol{\theta}_k$ in (S1), when moving from window $k-1$ to window k, as well as the required recursive update rules for $\mathbf{A}_k$ and $\mathbf{b}_k$. The parameter $\mathcal{S}_{FBS}$ in Algorithm 1 denotes the stopping condition for the FBS algorithm, which can be a maximum iteration number or a convergence criterion on the objective function.

Algorithm 1 Parameter Estimation in Dynamic Encoding and Decoding Models by Forward-Backward Splitting

Input: $\mathbf{y}_k$, $\mathbf{X}_k$, $\hat{\boldsymbol{\theta}}_{k-1}$, $\mathbf{A}_{k-1}$, $\mathbf{b}_{k-1}$, λ, γ, $\mathcal{S}_{FBS}$
Output: $\hat{\boldsymbol{\theta}}_k$, $\mathbf{A}_k$, $\mathbf{b}_k$

$\mathbf{A}_k = \lambda \mathbf{A}_{k-1} + \mathbf{X}_k^T \mathbf{X}_k$
$\mathbf{b}_k = \lambda \mathbf{b}_{k-1} - 2\mathbf{X}_k^T \mathbf{y}_k$
initialize $\boldsymbol{\theta}$ with $\hat{\boldsymbol{\theta}}_{k-1}$
while $\neg \mathcal{S}_{FBS}$ **do**
  choose stepsize τ
  $\mathbf{u} = \boldsymbol{\theta} - \tau \left(2\mathbf{A}_k \boldsymbol{\theta} + \mathbf{b}_k\right)$
  $\boldsymbol{\theta}_i = \mathrm{sign}(\mathbf{u}_i) \times \max\left\{|\mathbf{u}_i| - \gamma\tau, 0\right\}$, for each element of $\boldsymbol{\theta}$
end while
$\hat{\boldsymbol{\theta}}_k = \boldsymbol{\theta}$.

Remark 1. A proper step-size choice in Alg. 1 at each FBS iteration is crucial to the convergence of the algorithm. For a fixed step-size, it has been shown that $\tau < \frac{2}{L(\nabla f_k)}$ ensures the stability and convergence of the algorithm (Combettes and Pesquet, 2011), where $L(.)$ represents the Lipschitz constant, and f_k represents the log-likelihood term in (S1). Through standard Cauchy-Schwarz and triangle inequality manipulations, we can calculate the simple upper bound $L(\nabla f_k) \leq L_{\text{ub}} = 2\sum_{j=1}^{k} \lambda^{k-j} \, \mathrm{trace}\left\{\mathbf{X}_k^T \mathbf{X}_k\right\}$, implying that $\tau < \frac{2}{L_{\text{ub}}}$ ensures stability; however, this loose upper bound may decrease the convergence rate of the algorithm. Thus, it is more beneficial to ensure stability through backtracking and employing acceleration schemes such as adaptive step-size selection or the Nesterov's method (Goldstein et al., 2014). We have used the FASTA software package (Goldstein et al., 2014) available online at (Goldstein et al., 2015) in this work, which has built-in features for all the foregoing FBS step-size adjustment methods.

2 DYNAMIC STATE-SPACE MODEL: PARAMETER ESTIMATION

Recall that p_k denotes the probability of attending to speaker 1 at instance k for $k = 1, \ldots, K_A$. Although each k corresponds to a data window in time, we refer to it as an *instance* not to conflate it with the fixed-lag

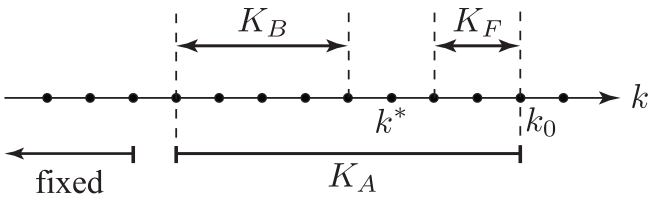

Figure S1. The parameters involved in state-space fixed-lag smoothing.

sliding window used for state estimation. The parameter K_A denotes the number of instances in fixed-lag smoothing as shown in Figure S1 (replaced from Figure 2 for completeness).

The linear state-space model which we apply on $\mathrm{logit}(p_k) = \ln\left(\frac{p_k}{1-p_k}\right)$, can be summarized as:

$$\begin{cases} p_k = \mathrm{P}\,(n_k\!=\!1) = 1 - \mathrm{P}\,(n_k\!=\!2) = \frac{1}{1+\exp(-z_k)} \\ z_k = c_0 z_{k-1} + w_k \\ w_k \sim \mathcal{N}(0, \eta_k) \\ \eta_k \sim \text{Inverse-Gamma}\,(a_0, b_0) \end{cases} \tag{S3}$$

Let $m_k^{(1)}$ and $m_k^{(2)}$ represent the attention markers and n_k represent a binary random variable taking values 1 or 2 depending on the attended speaker at instance k for $k = 1, \dots, K_A$. The observation equations of the state-space model, which relate the observed $m_{1:K_A}^{(1)}$ and $m_{1:K_A}^{(2)}$ to the hidden variables of the state-space model in Eq. (S3), can be summarized as:

$$\begin{cases} m_k^{(i)} \,\Big|\, n_k\!=\!i \sim \text{Log-Normal}\left(\rho^{(a)}, \mu^{(a)}\right), \quad i = 1, 2 \\ m_k^{(i)} \,\Big|\, n_k\!\neq\! i \sim \text{Log-Normal}\left(\rho^{(u)}, \mu^{(u)}\right), \quad i = 1, 2 \\ \rho^{(a)} \sim \text{Gamma}\left(\alpha_0^{(a)}, \beta_0^{(a)}\right), \quad \mu^{(a)} \,\Big|\, \rho^{(a)} \sim \mathcal{N}\left(\mu_0^{(a)}, \rho^{(a)}\right) \\ \rho^{(u)} \sim \text{Gamma}\left(\alpha_0^{(u)}, \beta_0^{(u)}\right), \quad \mu^{(u)} \,\Big|\, \rho^{(u)} \sim \mathcal{N}\left(\mu_0^{(u)}, \rho^{(u)}\right) \end{cases} \tag{S4}$$

The parameters of the state-space model are, therefore, $\boldsymbol{\Omega} = \left\{z_{1:K_A}, \eta_{1:K_A}, \rho^{(a)}, \mu^{(a)}, \rho^{(u)}, \mu^{(u)}\right\}$, which have to be inferred from $m_{1:K_A}^{(1)}$ and $m_{1:K_A}^{(2)}$. For notational simplicity, hereafter we use the boldface version of a variable to denote a vector containing all its instances, e.g., $\boldsymbol{z} := z_{1:K_A}$ and $\boldsymbol{m}^{(i)} := m_{1:K_A}^{(i)}$ for $i = 1, 2$.

The inference problem for $\boldsymbol{\Omega}$ can be expressed as:

$$\widehat{\boldsymbol{\Omega}} = \underset{\boldsymbol{\Omega}}{\arg\max}\ \ln \mathrm{P}\left(\boldsymbol{\Omega} \,\middle|\, \boldsymbol{m}^{(1)}, \boldsymbol{m}^{(2)}\right) = \underset{\boldsymbol{\Omega}}{\arg\max}\ \ln \mathrm{P}\left(\boldsymbol{m}^{(1)}, \boldsymbol{m}^{(2)} \,\middle|\, \boldsymbol{\Omega}\right) + \ln \mathrm{P}\left(\boldsymbol{\Omega}\right), \tag{S5}$$

where the log-likelihood and the log-prior are respectively expanded as:

$$\ln \mathrm{P}\left(\boldsymbol{m}^{(1)}, \boldsymbol{m}^{(2)} \mid \boldsymbol{\Omega}\right) = \ln\left(\sum_{n_{1:K_A}} \sum_{k=1}^{K_A} p_k \, \mathrm{P}\left(m_k^{(1)} \mid n_k, \boldsymbol{\Omega}\right) \mathrm{P}\left(m_k^{(2)} \mid n_k, \boldsymbol{\Omega}\right)\right), \tag{S6}$$

$$\ln \mathrm{P}(\boldsymbol{\Omega}) = \ln \mathrm{P}\left(\rho^{(a)}, \mu^{(a)}\right) + \ln \mathrm{P}\left(\rho^{(u)}, \mu^{(u)}\right) + \underbrace{\sum_{k=1}^{K_A}\left[-\frac{1}{2}\ln \eta_k - \frac{(z_k - c_0 z_{k-1})^2}{2\eta_k} + \ln \mathrm{P}(\eta_k)\right]}_{\ln \mathrm{P}(\boldsymbol{z},\boldsymbol{\eta})} + \mathsf{cnst}. \tag{S7}$$

Similar to the treatment in (Akram et al., 2016), we use an Expectation Maximization (EM) algorithm with $\boldsymbol{n}$ as the latent variables to infer $\boldsymbol{\Omega}$. Note that the optimization problem in (S5) is non-convex in general; thus, the choice of initial conditions and hyperparameters for priors are important for reaching a desirable local maximum. Having the estimate $\widehat{\boldsymbol{\Omega}}^{(\ell)}$ for $\boldsymbol{\Omega}$ at the ℓth EM iteration, we will next derive the E-step and M-step of the $(\ell+1)^{\text{th}}$ EM iteration.

2.1 The E-step

In the E-step, the surrogate function $Q\left(\boldsymbol{\Omega} \mid \widehat{\boldsymbol{\Omega}}^{(\ell)}\right)$ is calculated as:

$$Q\left(\boldsymbol{\Omega} \mid \widehat{\boldsymbol{\Omega}}^{(\ell)}\right) = \frac{1}{K_A} \underbrace{\mathbb{E}\left\{\ln \mathrm{P}\left(\boldsymbol{m}^{(1)}, \boldsymbol{m}^{(2)}, \boldsymbol{n} \mid \boldsymbol{\Omega}\right)\right\}}_{\mathcal{A}} + \ln \mathrm{P}(\boldsymbol{\Omega}), \tag{S8}$$

where the expectation of the *complete* log-likelihood $\ln \mathrm{P}\left(\boldsymbol{m}^{(1)}, \boldsymbol{m}^{(2)}, \boldsymbol{n} \mid \boldsymbol{\Omega}\right)$ needs to be calculated with respect to $\boldsymbol{n}$ given $\boldsymbol{m}^{(1)}, \boldsymbol{m}^{(2)}, \widehat{\boldsymbol{\Omega}}^{(\ell)}$. For notational simplicity, hereafter we drop the $\boldsymbol{n} \mid \boldsymbol{m}^{(1)}, \boldsymbol{m}^{(2)}, \widehat{\boldsymbol{\Omega}}^{(\ell)}$ subscript of the conditional expectations.

We have used a *normalized* version of the log-likelihood in Eq. (S8) for two reasons. First, the window length K_A is a hyperparameter in our framework, which we can modify to find the optimal trade-off between the dimensionality of the state-space and history-dependence of the model. Thus, to change the window length for fixed priors, it is important to normalize the contribution of the log-likelihood in (S8). Second, as noted before, we have a non-convex inference problem, which makes the resulting local maximum dependent on the conjugate priors used. We can use samples of $m_k^{(i)}$'s to estimate the attended and the unattended Log-Normal distributions and tune the hyperparameters to these distributions. By normalizing the log-likelihood term, we are enforcing informative and empirical prior distributions which would guide the inference procedure towards a plausible local maximum. For instance, for the correlation-based attention marker, we expect that a plausible solution would result in the attended Log-Normal distribution being concentrated around larger correlation values compared to the unattended distribution. Nevertheless, the forthcoming derivations can be carried out without the normalization factor $1/K_A$ in a similar fashion.

Let $\mathbb{I}_u(v)$ represent the indicator function, i.e., it is equal to one if $v = u$ and zero otherwise. Conditioning on $\boldsymbol{n}$ and using the conditional independence of $\boldsymbol{m}^{(1)}$ and $\boldsymbol{m}^{(2)}$ given $\boldsymbol{n}$ and $\boldsymbol{\Omega}$, the expected log-likelihood $\mathcal{A}$ in (S8) can be simplified as:

$$
\begin{aligned}
\mathcal{A} &= \sum_{i=1}^{2} \mathbb{E}\left\{\ln \mathrm{P}\left(\boldsymbol{m}^{(i)} \mid \boldsymbol{n}, \boldsymbol{\Omega}\right)\right\} + \mathbb{E}\left\{\ln \mathrm{P}\left(\boldsymbol{n} \mid \boldsymbol{\Omega}\right)\right\} \\
&= \sum_{k=1}^{K_A} \left[\sum_{i=1}^{2} \mathbb{E}\left\{\ln \mathrm{P}\left(m_k^{(i)} \mid n_k, \boldsymbol{\Omega}\right)\right\} + \mathbb{E}\left\{\ln \mathrm{P}\left(n_k \mid \boldsymbol{\Omega}\right)\right\}\right] \\
&= \sum_{k=1}^{K_A} \left[\sum_{i=1}^{2}\sum_{j=1}^{2} \mathbb{E}\left\{\mathbb{I}_j(n_k)\right\} \ln \mathrm{P}\left(m_k^{(i)} \mid n_k = j, \boldsymbol{\Omega}\right) + \underbrace{\mathbb{E}\left\{\mathbb{I}_1(n_k)\right\} p_k + \mathbb{E}\left\{\mathbb{I}_2(n_k)\right\}(1-p_k)}_{\mathbb{E}\left\{\ln \mathrm{P}\left(n_k \mid \boldsymbol{\Omega}\right)\right\}}\right].
\end{aligned}
\tag{S9}
$$

Note that $m_k^{(i)} \mid n_k, \boldsymbol{\Omega}$ pertains to either the attended or unattended Log-Normal distributions in Eq. (S4) depending on the values of i and n_k. Considering that the n_k's are binary random variables and the expectations are with respect to $\boldsymbol{n} \mid \boldsymbol{m}^{(1)}, \boldsymbol{m}^{(2)}, \widehat{\boldsymbol{\Omega}}^{(\ell)}$, the term $\mathbb{E}\left\{\mathbb{I}_j(n_k)\right\}$ can be computed for $j = 1, 2$ using Bayes' rule and conditional independence as:

$$
\begin{aligned}
\mathbb{E}\left\{\mathbb{I}_j(n_k)\right\} &= \mathrm{P}\left(n_k = j \mid \boldsymbol{m}^{(1)}, \boldsymbol{m}^{(2)}, \widehat{\boldsymbol{\Omega}}^{(\ell)}\right) \\
&= \mathrm{P}\left(n_k = j \mid m_k^{(1)}, m_k^{(2)}, \widehat{\boldsymbol{\Omega}}^{(\ell)}\right) \\
&= \frac{\mathrm{P}\left(m_k^{(1)}, m_k^{(2)} \mid n_k = j, \widehat{\boldsymbol{\Omega}}^{(\ell)}\right) \mathrm{P}\left(n_k = j \mid \widehat{\boldsymbol{\Omega}}^{(\ell)}\right)}{\mathrm{P}\left(m_k^{(1)}, m_k^{(2)} \mid \widehat{\boldsymbol{\Omega}}^{(\ell)}\right)} \\
&= \frac{\mathrm{P}\left(m_k^{(1)} \mid n_k = j, \widehat{\boldsymbol{\Omega}}^{(\ell)}\right) \mathrm{P}\left(m_k^{(2)} \mid n_k = j, \widehat{\boldsymbol{\Omega}}^{(\ell)}\right) \mathrm{P}\left(n_k = j \mid \widehat{\boldsymbol{\Omega}}^{(\ell)}\right)}{\sum_{n_k} \mathrm{P}\left(m_k^{(1)} \mid n_k, \widehat{\boldsymbol{\Omega}}^{(\ell)}\right) \mathrm{P}\left(m_k^{(2)} \mid n_k, \widehat{\boldsymbol{\Omega}}^{(\ell)}\right) \mathrm{P}\left(n_k \mid \widehat{\boldsymbol{\Omega}}^{(\ell)}\right)}.
\end{aligned}
\tag{S10}
$$

The parameters of the Log-Normal distributions for $m_k^{(i)} \mid n_k, \widehat{\boldsymbol{\Omega}}^{(\ell)}$ are determined from the estimated $\left(\rho^{(a)}, \mu^{(a)}, \rho^{(u)}, \mu^{(u)}\right)$ in the previous EM iteration, i.e., $\widehat{\boldsymbol{\Omega}}^{(\ell)}$. Also, $\mathrm{P}\left(n_k \mid \widehat{\boldsymbol{\Omega}}^{(\ell)}\right) = \frac{1}{1+\exp\left(-\hat{z}_k^{(\ell)}\right)}$ in (S10), where $\hat{z}_k^{(\ell)}$ is the estimate of z_k from the previous EM iteration. Note that $\mathbb{E}\left\{\mathbb{I}_1(n_k)\right\} = 1 - \mathbb{E}\left\{\mathbb{I}_2(n_k)\right\}$ as n_k is a binary random variable. Defining $\epsilon_k^{(\ell)} := \mathbb{E}\left\{\mathbb{I}_1(n_k)\right\}$ with the expectation over $n_k \mid m_k^{(1)}, m_k^{(2)}, \widehat{\boldsymbol{\Omega}}^{(\ell)}$, we can conclude the E-step by simplifying $Q\left(\boldsymbol{\Omega} \mid \widehat{\boldsymbol{\Omega}}^{(\ell)}\right)$ in Eq. (S8) as:

$$Q\left(\boldsymbol{\Omega} \mid \widehat{\boldsymbol{\Omega}}^{(\ell)}\right) = \sum_{k=1}^{K_A} \frac{1}{2K_A} \Bigg\{ -\rho^{(a)} \left[\epsilon_k^{(\ell)} \left(\ln m_k^{(1)} - \mu^{(a)}\right)^2 + \left(1-\epsilon_k^{(\ell)}\right) \left(\ln m_k^{(2)} - \mu^{(a)}\right)^2 \right]$$
$$- \rho^{(u)} \left[\left(1-\epsilon_k^{(\ell)}\right) \left(\ln m_k^{(1)} - \mu^{(u)}\right)^2 + \epsilon_k^{(\ell)} \left(\ln m_k^{(2)} - \mu^{(u)}\right)^2 \right]$$
$$+ \ln \rho^{(a)} + \ln \rho^{(u)} \Bigg\}$$
$$- \rho^{(a)} \left[\beta_0^{(a)} + 0.5 \left(\mu^{(a)} - \mu_0^{(a)}\right)^2 \right] + \left(\alpha_0^{(a)} - 0.5\right) \ln \rho^{(a)}$$
$$- \rho^{(u)} \left[\beta_0^{(u)} + 0.5 \left(\mu^{(u)} - \mu_0^{(u)}\right)^2 \right] + \left(\alpha_0^{(u)} - 0.5\right) \ln \rho^{(u)}$$
$$+ \sum_{k=1}^{K_A} \left\{ \epsilon_k^{(\ell)} p_k + \left(1-\epsilon_k^{(\ell)}\right)(1-p_k) - (a_0 + 1.5) \ln \eta_k - \frac{1}{\eta_k} \left[b_0 + 0.5(z_k - c_0 z_{k-1})^2 \right] \right\}$$
$$+ \mathsf{cnst}. \tag{S11}$$

where the $\mathsf{cnst}.$ term includes all the terms that are independent of $\boldsymbol{\Omega}$.

2.2 The M Step

In the M step, we maximize $Q\left(\boldsymbol{\Omega} \mid \widehat{\boldsymbol{\Omega}}^{(\ell)}\right)$ in Eq. (S11) with respect to $\boldsymbol{\Omega}$. The maximizers form the parameter updates for the $(\ell{+}1)^{\text{th}}$ EM iteration. As we observe in Eq. (S11), having $\boldsymbol{n}$ as the latent variables separates the terms in $Q\left(\boldsymbol{\Omega} \mid \widehat{\boldsymbol{\Omega}}^{(\ell)}\right)$ depending on the distribution parameters, i.e., $\left(\rho^{(a)}, \mu^{(a)}, \rho^{(u)}, \mu^{(u)}\right)$, and the terms depending on the state-space parameters, i.e., $\boldsymbol{z}$ and $\boldsymbol{\eta}$. The derivation of the update rules for the distribution parameters is straightforward through taking the derivatives of $Q\left(\boldsymbol{\Omega} \mid \widehat{\boldsymbol{\Omega}}^{(\ell)}\right)$ and solving for their joint zero-crossings. Consequently, the closed-form formulas for the distribution parameters maximizing $Q\left(\boldsymbol{\Omega} \mid \widehat{\boldsymbol{\Omega}}^{(\ell)}\right)$ can be expressed as:

$$\mu^{(a)^*} = \frac{1}{2} \left\{ \mu_0^{(a)} + \frac{1}{K_A} \sum_{k=1}^{K_A} \left[\epsilon_k^{(\ell)} \ln m_k^{(1)} + \left(1-\epsilon_k^{(\ell)}\right) \ln m_k^{(2)} \right] \right\}, \tag{S12}$$

$$\mu^{(u)^*} = \frac{1}{2} \left\{ \mu_0^{(u)} + \frac{1}{K_A} \sum_{k=1}^{K_A} \left[\left(1-\epsilon_k^{(\ell)}\right) \ln m_k^{(1)} + \epsilon_k^{(\ell)} \ln m_k^{(2)} \right] \right\}, \tag{S13}$$

$$\rho^{(a)*} = \frac{2K_A\alpha_0^{(a)}}{\sum_{k=1}^{K_A}\left[\epsilon_k^{(\ell)}\left(\ln m_k^{(1)} - \mu^{(a)*}\right)^2 + \left(1-\epsilon_k^{(\ell)}\right)\left(\ln m_k^{(2)} - \mu^{(a)*}\right)^2\right] + K_A\left[2\beta_0^{(a)} + \left(\mu^{(a)*} - \mu_0^{(a)}\right)^2\right]}, \quad \text{(S14)}$$

$$\rho^{(u)*} = \frac{2K_A\alpha_0^{(u)}}{\sum_{k=1}^{K_A}\left[\left(1-\epsilon_k^{(\ell)}\right)\left(\ln m_k^{(1)} - \mu^{(u)*}\right)^2 + \epsilon_k^{(\ell)}\left(\ln m_k^{(2)} - \mu^{(u)*}\right)^2\right] + K_A\left[2\beta_0^{(u)} + \left(\mu^{(u)*} - \mu_0^{(u)}\right)^2\right]}, \quad \text{(S15)}$$

where $\left(\rho^{(a)^*}, \mu^{(a)^*}, \rho^{(u)^*}, \mu^{(u)^*}\right)$ will be the updated distribution parameters in $\widehat{\mathbf{\Omega}}^{(\ell+1)}$.

The next step is to maximize $Q\left(\mathbf{\Omega} \mid \widehat{\mathbf{\Omega}}^{(\ell)}\right)$ with respect to $\boldsymbol{z}$ and $\boldsymbol{\eta}$. Note that this joint maximization is non-convex in general. Consider the following state-space model with parameters $(\boldsymbol{z'}, \boldsymbol{\eta'})$ and binary observations $\boldsymbol{n'}$.

$$\begin{cases} n'_k \sim \text{Bernoulli}\left(\frac{1}{1+\exp(-z'_k)}\right) \\ z'_k = c_0 z'_{k-1} + w'_k \\ w'_k \sim \mathcal{N}(0, \eta'_k) \\ \eta'_k \sim \text{Inverse-Gamma}\,(a_0, b_0) \end{cases} \quad \text{(S16)}$$

For the inference problem in (S16), the log-posterior can be expressed as:

$$\underset{\boldsymbol{z'},\boldsymbol{\eta'}}{\arg\max} \ln \mathrm{P}\left(\boldsymbol{z'}, \boldsymbol{\eta'} \mid \boldsymbol{n'}\right) = \underset{\boldsymbol{z'},\boldsymbol{\eta'}}{\arg\max}\left[\ln \mathrm{P}\left(\boldsymbol{\eta'} \mid \boldsymbol{n'}\right) + \mathrm{P}\left(\boldsymbol{z'} \mid \boldsymbol{\eta'}, \boldsymbol{n'}\right)\right]. \quad \text{(S17)}$$

If we replace the observations n'_k in (S17) with $\epsilon_k^{(\ell)}$, for $k = 1, 2, \ldots, K_A$, the inference problem becomes equivalent to maximizing $Q\left(\mathbf{\Omega} \mid \widehat{\mathbf{\Omega}}^{(\ell)}\right)$ in (S11) with respect to $\boldsymbol{z}$ and $\boldsymbol{\eta}$.

In (Smith and Brown, 2003; Smith et al., 2004), the inference of the parameters in (S16) has been carried out through the EM algorithm, where in each iteration, a Kalman filtering and smoothing algorithm has been employed together with Gaussian approximations. Similar to (Akram et al., 2016), we refer to this EM algorithm as the inner EM not to confuse it with the EM algorithm we have already adopted, which we call the outer EM hereafter. The basic idea behind the inner EM is to approximate the solutions to (S17) as:

$$\begin{cases} \boldsymbol{\eta'}^* = \arg\max_{\boldsymbol{\eta'}} \mathrm{P}\left(\boldsymbol{\eta'} \mid \boldsymbol{n'}\right) \\ \boldsymbol{z'}^* = \arg\max_{\boldsymbol{z'}} \mathrm{P}\left(\boldsymbol{z'} \mid \boldsymbol{\eta'}^*, \boldsymbol{n'}\right) \end{cases}, \quad \text{(S18)}$$

where $\boldsymbol{\eta}'^{*}$ are estimated through the inner EM with $\boldsymbol{z}'$ as the latent variables, and $\boldsymbol{z}'^{*}$ are just the result of a Kalman filtering and smoothing algorithm in (S16) for $\boldsymbol{\eta}' = \boldsymbol{\eta}'^{*}$.

In order to make the inference procedure suitable for real-time implementation, we can avoid the inner EM and instead use crude estimates of $\boldsymbol{\eta}'^{*}$ in (S18). Note that $\epsilon_k^{(\ell)}$, which acts as the observation n'_k in (S16) for $k = 1, 2, \ldots, K_A$, is equal to $\mathrm{P}\left(n_k = 1 \mid m_k^{(1)}, m_k^{(2)}, \widehat{\boldsymbol{\Omega}}^{(\ell)}\right)$ calculated as in (S10). Assuming that $\epsilon_k^{(\ell)} \approx \mathrm{P}\left(n'_k = 1\right) = \frac{1}{1+\exp(-z'_k)}$, in the ℓth outer EM iteration, we can consider $\left[\text{logit}\left(\epsilon_k^{(\ell)}\right) - c_0\,\text{logit}\left(\epsilon_{k-1}^{(\ell)}\right)\right]$ as a sample of $\mathcal{N}\left(0, {\eta'}_k\right)$. Therefore, considering the Inverse-Gamma prior, a crude estimate for ${\eta'}_k^{*}$ can be calculated for $k = 1, 2, \ldots, K_A$ as:

$$ {\eta'}_k^{*} = \frac{2b_0 + \left[\text{logit}\left(\epsilon_k^{(\ell)}\right) - c_0\,\text{logit}\left(\epsilon_{k-1}^{(\ell)}\right)\right]^2}{2a_0 - 1}. \tag{S19} $$

If K_A is small enough, we can simplify the state-space model of (S16) by assuming a single variance, i.e., $\eta' = \eta'_k$ for $k = 1, 2, \ldots, K_A$, and using an estimate similar to (S19) for ${\eta'}^{*}$. However, in this model, the crude estimate would be more reliable as it is based on K_A samples rather than a single sample. Considering a normalized log-likelihood and the same Inverse-Gamma prior on η', the estimate for ${\eta'}^{*}$ can be computed as:

$$ {\eta'}^{*} = \frac{2b_0 + \frac{1}{K_A}\sum_{k=1}^{K_A}\left[\text{logit}\left(\epsilon_k^{(\ell)}\right) - c_0\,\text{logit}\left(\epsilon_{k-1}^{(\ell)}\right)\right]^2}{2a_0 - 1}. \tag{S20} $$

After estimating ${\eta'}_k^{*}$ in (S19) for $k = 1, 2, \ldots, K_A$, or ${\eta'}^{*}$ in (S20), we can proceed as before to estimate $\boldsymbol{z}'^{*}$, i.e., using a Kalman filtering and smoothing algorithm with Gaussian approximations to estimate $\boldsymbol{z}'^{*}$ in (S18). These estimates, namely $\boldsymbol{z}^{*}$ and $\boldsymbol{\eta}^{*}$, form approximate solutions for $\boldsymbol{z}$ and $\boldsymbol{\eta}$ in the original problem of maximizing $Q(\boldsymbol{\Omega} \mid \widehat{\boldsymbol{\Omega}}^{(\ell)})$ in (S11) with respect to the state-space parameters.

Next, we discuss the details of the inner EM algorithm, as in (Akram et al., 2016), used to solve for $\boldsymbol{z}'$ and $\boldsymbol{\eta}'$ in (S16). As mentioned before, the idea is to use an EM algorithm together with Gaussian approximations to maximize $\mathrm{P}\left(\boldsymbol{\eta}' \mid \boldsymbol{n}'\right)$, and then maximize the likelihood of $\boldsymbol{z}'$ with respect to the observations and estimated variances. Considering $\boldsymbol{z}'$ as the latent variables, the surrogate function $Q(\boldsymbol{\eta}' | \widehat{\boldsymbol{\eta}'}^{(\ell)})$ at ℓ^{th} EM iteration is calculated as:

$$ \begin{aligned} Q\left(\boldsymbol{\eta}' | \widehat{\boldsymbol{\eta}'}^{(\ell)}\right) &= \mathbb{E}\left\{\ln \mathrm{P}\left(\boldsymbol{n}', \boldsymbol{z}' \mid \boldsymbol{\eta}'\right)\right\} + \ln \mathrm{P}(\boldsymbol{\eta}') \\ &= \sum_{k=1}^{K_A}\left[\frac{\mathbb{E}\left\{(z'_k - c_0 z'_{k-1})^2\right\} + 2b_0}{2\eta'_k} + (a_0 + 1.5)\ln \eta'_k\right] + \text{cnst.}, \end{aligned} \tag{S21} $$

where the expectations are with respect to $\boldsymbol{z}' \mid \boldsymbol{n}', \widehat{\boldsymbol{\eta}'}^{(\ell)}$, and the cnst. term contains all the terms that are independent of $\boldsymbol{\eta}'$.

In the M-step of the inner EM algorithm, $Q\left(\boldsymbol{\eta}' \middle| \widehat{\boldsymbol{\eta}'}^{(\ell)}\right)$ is maximized with respect to $\boldsymbol{\eta}'$ to calculate the updated variances for the next EM iteration. Taking the derivative of (S21) with respect to $\boldsymbol{\eta}'$ and equating it to zero results in the following update rule for $\widehat{\boldsymbol{\eta}'}^{(\ell+1)}$:

$$\begin{aligned}\widehat{\eta'}_k^{(\ell+1)} &= \frac{1}{2a_0+3}\left[\mathbb{E}\left\{(z'_k - c_0 z'_{k-1})^2\right\} + 2b_0\right] \\ &= \frac{1}{2a_0+3}\left[\mathbb{E}\left\{{z'_k}^2\right\} + c_0^2\mathbb{E}\left\{{z'_{k-1}}^2\right\} - 2c_0\mathbb{E}\left\{z'_k z'_{k-1}\right\} + 2b_0\right] \\ &= \frac{1}{2a_0+3}\Big[\sigma^2_{k|K_A} + \bar{z}^2_{k|K_A} + c_0^2\sigma^2_{k-1|K_A} + c_0^2\bar{z}^2_{k-1|K_A} - 2c_0\sigma^2_{k,k-1|K_A} \\ &\qquad -2c_0\bar{z}_{k|K_A}\bar{z}_{k-1|K_A} + 2b_0\Big],\end{aligned} \tag{S22}$$

where the parameters $\bar{z}_{k|K_A}$ and $\sigma^2_{k|K_A}$ in Eq. (S22) are respectively the mean and the variance of $z'_k \mid \boldsymbol{n}', \widehat{\boldsymbol{\eta}'}^{(\ell)}$.

If we consider the Gaussian approximation $\mathcal{N}\left(\bar{z}_{k_1|k_2}, \sigma^2_{k_1|k_2}\right)$ to the density $z'_{k_1} | n'_{1:k_2}, \widehat{\boldsymbol{\eta}'}^{(\ell)}$ for $1 \leq k_1 \leq k_2 \leq K_A$, these parameters can be computed in a forward and backward pass similar to the conventional Kalman filtering and smoothing algorithms. The corresponding filtering equations for $1 \leq k \leq K_A$ are summarized as:

$$\begin{cases} \bar{z}_{k|k-1} = c_0 \bar{z}_{k-1|k-1} \\ \sigma^2_{k|k-1} = c_0^2 \sigma^2_{k-1|k-1} + {\eta'}_k^{(l)} \\ \bar{z}_{k|k} = \bar{z}_{k|k-1} + \sigma^2_{k|k-1}\left[n'_k - \frac{\exp(\bar{z}_{k|k})}{1+\exp(\bar{z}_{k|k})}\right] \\ \sigma^2_{k|k} = \left[\frac{1}{\sigma^2_{k|k-1}} + \frac{\exp(\bar{z}_{k|k})}{\left(1+\exp(\bar{z}_{k|k})\right)^2}\right]^{-1} \end{cases} \tag{S23}$$

Note that the third equation in (S23) is a non-linear equation whose solution can be approximated through standard approaches such as the Newton's method. The last two equations in (S23) come from the Gaussian approximation: assuming that $z'_{k-1} \mid n'_{1:k-1}, \widehat{\boldsymbol{\eta}'}^{(\ell)} \backsim \mathcal{N}\left(\bar{z}_{k-1|k-1}, \sigma^2_{k-1|k-1}\right)$ we calculate the Gaussian approximation for $z'_k \mid n'_{1:k}, \widehat{\boldsymbol{\eta}'}^{(\ell)}$. The mean of the Gaussian approximation $\bar{z}_{k|k}$ is calculated as the mode of $\ln \mathrm{P}\left(z'_k \mid n'_{1:k}, \widehat{\boldsymbol{\eta}'}^{(\ell)}\right)$, and its variance $\sigma^2_{k|k}$ is computed as the negative inverse Hessian of $\ln \mathrm{P}\left(z'_k \mid n'_{1:k}, \widehat{\boldsymbol{\eta}'}^{(\ell)}\right)$ evaluated at the estimated mean $\bar{z}_{k|k}$ (Tanner, 1991). The smoothing equations are the same as those used for fixed interval smoothing. Therefore, for $1 \leq k \leq K_A - 1$, we have:

$$\begin{cases} s_k = \sigma^2_{k|k} \Big/ \sigma^2_{k+1|k} \\ \bar{z}_{k|K_A} = \bar{z}_{k|k} + s_k \left(\bar{z}_{k+1|K_A} - \bar{z}_{k+1|k} \right) \\ \sigma^2_{k|K_A} = \sigma^2_{k|k} + s_k^2 \left(\sigma^2_{k+1|K_A} - \sigma^2_{k+1|k} \right) \end{cases} \tag{S24}$$

The $\sigma^2_{k,k-1|K_A}$ term in (S22) is a lagged covariance term that can be computed using the covariance smoothing algorithm (De Jong and Mackinnon, 1988):

$$\sigma^2_{k,k-1|K_A} = \mathrm{Cov}\left\{ z'_k, z'_{k-1} \mid \boldsymbol{n}', \widehat{\boldsymbol{\eta}'}^{(\ell)} \right\} = \frac{\sigma^2_{k-1|k-1}\sigma^2_{k|K_A}}{\sigma^2_{k|k-1}}. \tag{S25}$$

Having calculated the variances $\boldsymbol{\eta}'^{*}$ from the inner EM algorithm, $\boldsymbol{z}'^{*}$ can be estimated using a single forward and backward pass for $\boldsymbol{\eta}' = \boldsymbol{\eta}'^{*}$, similar to that used in the inner EM algorithm. In summary, we have transformed the problem of maximizing (S11) with respect to $\boldsymbol{z}$ and $\boldsymbol{\eta}$ into inferring $\boldsymbol{z}'$ and $\boldsymbol{\eta}'$ in (S16) by identifying n'_k with $\epsilon_k^{(l)}$ for $k = 1, \ldots, K_A$. We have then solved the latter problem through an EM algorithm combined with Gaussian approximations and Kalman filtering and smoothing. Therefore, we have $\boldsymbol{z}^* = \boldsymbol{z}'^*$ and $\boldsymbol{\eta}^* = \boldsymbol{\eta}'^*$ in the original problem.

Algorithm 2 Parameter Estimation in Dynamic State-Space Model

Input: $m^{(1)}_{1:K_A}$, $m^{(2)}_{1:K_A}$, $\alpha_0^{(a)}$, $\alpha_0^{(u)}$, $\beta_0^{(a)}$, $\beta_0^{(u)}$, $\mu_0^{(a)}$, $\mu_0^{(u)}$, a_0, b_0, $\mathcal{S}_{EM}$
Output: $\widehat{\boldsymbol{\Omega}} = \left\{ \hat{z}_{1:K_A}, \hat{\eta}_{1:K_A}, \hat{\rho}^{(a)}, \hat{\mu}^{(a)}, \hat{\rho}^{(u)}, \hat{\mu}^{(u)} \right\}$

Set $\widehat{\boldsymbol{\Omega}}^{(0)}$ as the initialization for state-space model parameter set based on estimates in the previous instance
$\ell = 0$
while $\neg\mathcal{S}_{EM}$ **do**
    calculate $\epsilon^{(\ell)}_{1:K_A}$ using (S10)
    update the parameters of the Log-Normal distributions, i.e., $\mu^{(a)}$, $\mu^{(u)}$, $\rho^{(a)}$, $\rho^{(u)}$, based on equations (S12), (S13), (S14), and (S15) respectively
    update the state-space variances, i.e., $\eta_{1:K_A}$, using the inner-EM algorithm or the crude estimates in equations (S19) and (S20)
    update the hidden states in the state-space model, i.e., $z_{1:K_A}$, using a Kalman filtering and smoothing algorithm with Gaussian approximations
    set $\widehat{\boldsymbol{\Omega}}^{(\ell+1)}$ as the updated parameter set including the updated distribution parameters, variances, and hidden states in the state-space model
    $\ell \leftarrow \ell + 1$
end while
$\widehat{\boldsymbol{\Omega}} = \widehat{\boldsymbol{\Omega}}^{(\ell)}$.

Algorithm 2 summarizes the overall inference procedure within a fixed-lag window of length K_A. Going back to Fig. S1, copied from the paper, we assume $k = k_0$ is the current instance and the

goal is to infer the attentional state at instance $k = k_0 - K_F$ based on the attention markers within the window indexed from 1 to K_A, given by $m_k^{(i)}$ for $i = 1, 2$ and $k = 1, \ldots, K_A$. We initialize the state-space model parameter set $\mathbf{\Omega}$ using the estimates at the previous instance, and the output of Algorithm 2, i.e., $\widehat{\mathbf{\Omega}}$, is used for initialization in the next instance. Defining $f(.)$ as the sigmoid function, $f\left(\hat{z}_{K_A - K_F}\right)$ determines the estimated probability of attending to speaker 1 at $k = k_0 - K_F$, and $\left[f\left(\hat{z}_{K_A-K_F} - 1.65\hat{\sigma}^2_{K_A-K_F|K_A}\right), f\left(\hat{z}_{K_A-K_F} + 1.65\hat{\sigma}^2_{K_A-K_F|K_A}\right)\right]$ represents the 90% confidence intervals of this estimate, where $\hat{\sigma}^2_{K_A-K_F|K_A}$ represents the inferred variance of $\hat{z}_{K_A-K_F}$ calculated through the discussed Gaussian approximations. The parameter $\mathcal{S}_{EM}$ in Algorithm 2 is a stopping condition for the outer EM, which can be a limit on the number of iterations.

3 SMOOTHING EFFECT OF STATE-SPACE MODELING

In this section, we discuss the smoothing effect of the proposed state-space estimation and compare it with that of sliding Gaussian kernel smoothers. Recall that the Inverse-Gamma conjugate prior on η_k's in Eq. (S3) controls the degree of smoothing in the state-space model. If this prior favors smaller values of η_k's, the consecutive changes in z_k's and thereby p_k's will be smaller, which results in a larger smoothing effect. We tune the Inverse-Gamma prior through the hyperparameters a_0 and b_0 as in Eq. (S3) to match the auditory attention dynamics. Therefore, we expect that the corresponding smoothing effect will make the state-space estimates robust to the stochastic fluctuations in the attention markers, while capturing the attention switching instances with a small transition delay.

Fig. S2-A shows the output of the correlation-based attention marker in Case 2 of the simulation study in the main manuscript (row D of Fig. 4). The output of the real-time estimator with 1.5 s forward-lag (as in row F of Fig. 4) is shown in Fig. S2-B. We also consider two *non-causal* Gaussian kernel smoothers with the same delay of 1.5 s for fairness of comparison. Fig. S2-C and S2-E show the attention markers of Fig. S2-A convolved with the two Gaussian kernels, respectively. The two kernels are shown as insets in Fig. S2-C and S2-D. Gaussian kernel 1 in Fig. S2-C favors the current values of the attention marker while Gaussian kernel 2 in Fig. S2-D gives more weight to its future values.

Both kernels provide a clearer picture of the attentional state by smoothing out the stochastic fluctuations of Fig. S2-A. However, unlike the output of the state-space estimator, they do not provide statistically interpretable results. First, based on Figs. S2-C and -D, we can only obtain a binary decision on the attended speaker at each instance. The state-space estimates, however, provide a probabilistic measure of the attentional state as shown in Fig. S2-B, together with statistical confidence intervals. The red arrows in Fig. S2-C and -D mark instances where strong fluctuations in the attention markers result in misclassification. For instance, the smoothed markers with kernel 2 imply an attention switch earlier than the 30 s mark (upward arrow, Fig. S2-D). Such abrupt classification errors could be undesirable for applications such as BCI systems or smart hearing aids, as the devices need to modify their settings back and forth in a small time period. The state-space model prevents these instances of misclassification, thanks to the confidence intervals of the estimated p_k's (the middle arrows) which help rule out such false alarm events.

4 ENCODING MODEL SIMULATION

This section provides a simulated example to motivate our MEG analysis, in which we use an encoding model and take the M100 component of the Temporal Response Function (TRF) as the attention marker.

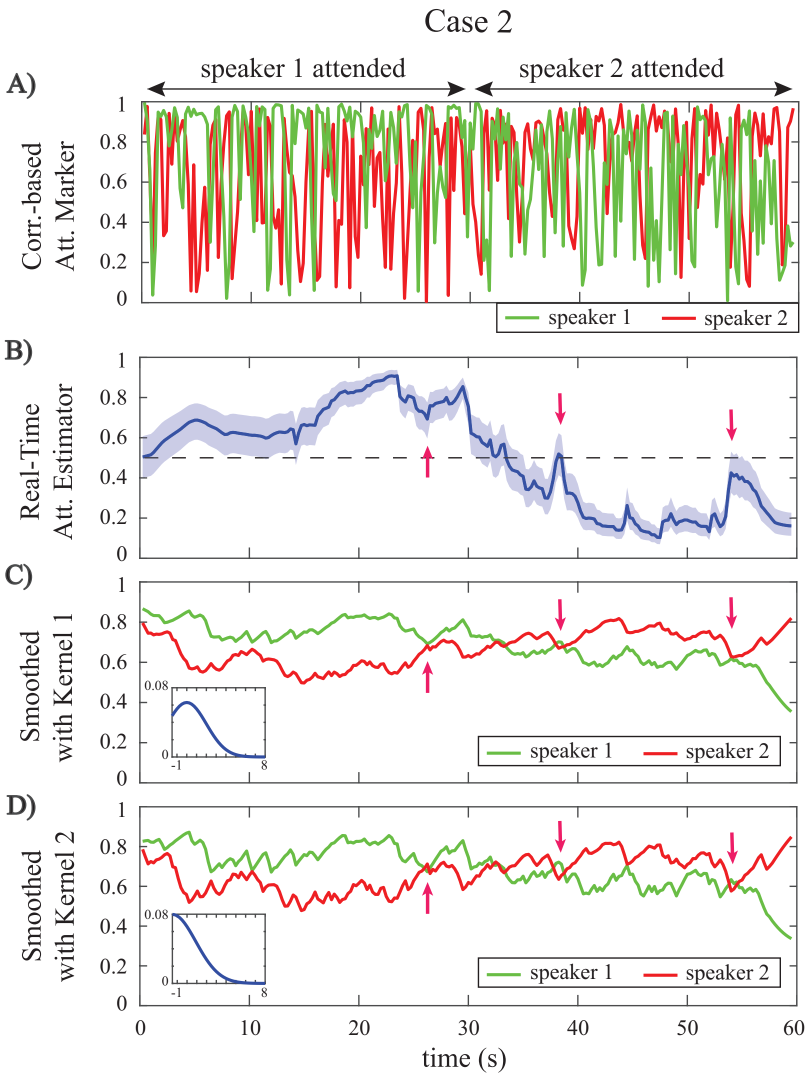

Figure S2. Smoothing effect of the state-space model in comparison to simple kernel smoothers: A) Output of the correlation-base attention marker corresponding to Case 2 of the simulation study in the main manuscript. B) Real-time estimator with 1.5 s forward-lag. C) Convolution of the correlation-based attention marker with Gaussian kernel 1 (shown as inset). D) Convolution of the correlation-based attention marker with Gaussian kernel 2 (shown as inset).

4.1 Simulation Settings

Consider the following generative model:

$$e_t = s_t^{(1)} * \tau_t^{(1)} + s_t^{(2)} * \tau_t^{(2)} + \mu + n_t, \tag{S26}$$

where e_t, $s_t^{(1)}$, and $s_t^{(2)}$ respectively denote the auditory component of the neural response, speech envelope for speaker 1, and speech envelope for speaker 2. We have used the same speech signals for $s_t^{(1)}$ and $s_t^{(2)}$ as in the EEG simulation, with the same sampling rate of $f_s = 200$ Hz. In the context of MEG processing, $\tau_t^{(1)}$ and $\tau_t^{(2)}$ are referred to as the TRF for speakers 1 and 2. We have set $\mu = 0.001$ as the unknown constant

mean and $n_t \overset{\text{iid}}{\sim} \mathcal{N}(0, 2.5 \times 10^{-7})$ as the observation noise. We assume an attention modulation effect on the M100 component of the TRFs.

Figure S3 shows two cases for the TRFs $\tau_t^{(1)}$ and $\tau_t^{(2)}$: In the left panels (case 1), there is a strong attention modulation effect on the M100 components, and in the right panels (case 2), this effect is weakened. In both cases, the attention is on speaker 1 during the $[0, 30)$ s interval and on speaker 2 during the $(30, 60]$ s interval. Also, we have considered a length of 0.4 s for the TRFs. Row B in Fig. S3 shows examples of the attended and the unattended TRFs for each of the two cases. In case 1, there is a large difference between the magnitude of the M100 components in the attended and the unattended TRFs, while in case 2, this difference is small compared to our estimation accuracy. We have also considered three higher latency components in the TRFs which are not modulated by the attentional state, similar to the M50 component. As shown in row A of Fig. S3, a zero-mean Gaussian i.i.d. noise is added to the TRF components as well. Note that similar to the EEG simulation, we have used a Gaussian kernel with the standard deviation of 10 ms to smooth the TRFs. This smoothness property is also observed in TRFs estimated from experimentally-recorded MEG signals (Ding and Simon, 2012a,b).

4.2 Parameter Selection

For the encoder estimation parameters in Algorithm 1, we have considered consecutive non-overlapping windows of length 0.25 s, i.e., $W = 50$, resulting in $K = 240$ instances, and we have assumed the same 0.4 s length for the TRFs, i.e., $L_e = 80$. We have chosen $\gamma = 0.005$ through cross-validation and $\lambda = 0.9167$, which results in an *effective* window length of 3 s for encoder estimation. Considering the smoothing Gaussian kernel used in the forward model, we have used the Gaussian dictionary matrix $\mathbf{G}_0 \in \mathbb{R}^{(L_e+1)\times(L_e+1)}$ for each speaker in the encoder estimation step to enforce smoothness in the TRFs. The dictionary columns consist of overlapping Gaussian kernels with the standard deviation of 10 ms, whose means cover the 0 s to 0.4 s lag with $T_s = 5$ ms increments. As a result, considering the simultaneous estimation of the two TRFs, the overall dictionary matrix would be $\mathbf{G} = \text{diag}\left(1, \mathbf{G_0}, \mathbf{G_0}\right)$.

We have used the FASTA package (Goldstein et al., 2014) with Nesterov's acceleration method to implement the forward-backward splitting algorithm. All the prior distribution parameters of the state-space models are set similar to the EEG simulation in the paper, where $a_0 = 2.008$, $b_0 = 0.2016$, and the prior parameters for the attended and unattended distributions were tuned based on a separate 15 s sample trial. For the real-time state-space estimator, we have used a sliding window of length 15 s with a fixed forward-lag of 1.5 s, i.e., $K_A = \lfloor 15 f_s / W \rfloor$ and $K_F = \lfloor 1.5 f_s / W \rfloor$. The sample trial for tuning the distribution parameters can be thought of as an initialization step for the estimator prior to its real-time application.

4.3 Estimation Results

Figure S4 shows the results of our estimation framework. Row A contains the estimated TRFs for the encoding model. The major components of the TRFs are retrieved in the estimates while the ℓ_1-norm penalty in Eq. (S1) has significantly denoised these components as compared with the original noisy versions in row A of Fig. S3. Row B in Fig. S4 displays the extracted magnitudes of the M100 components from the estimated TRFs at each instance. The attention marker in this case is defined as the magnitude of the M100 component, where the M100 component is calculated as the minimum value of the TRF estimate around the 100 ms lag. Notice that there is a significant statistical difference between the extracted M100 components for the attended and unattended speakers in case 1, while the estimated M100 components are highly variable in case 2 and do not show a strong attention modulation effect.

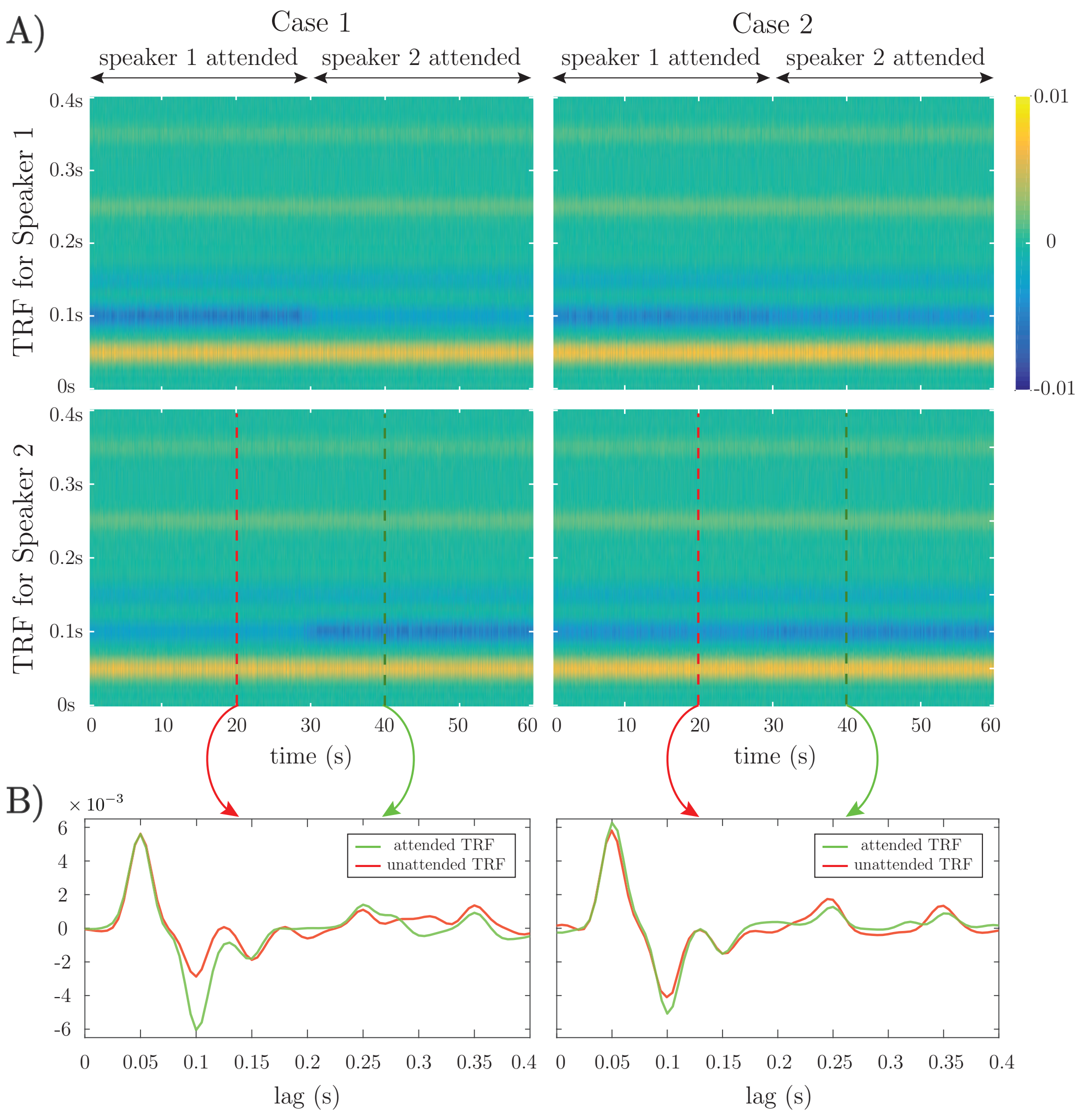

Figure S3. The TRFs $\tau_t^{(1)}$ and $\tau_t^{(2)}$ used for the simulation model in Eq. (S26). A) TRFs for case 1 (strong modulation in M100 components) and case 2 (weak modulation in M100 components). B) Snapshots of the attended and unattended TRFs for the two cases.

Rows C and D of Fig. S4 show the output of the batch-mode and real-time state-space estimators, respectively. In case 1, both the batch-mode and real-time estimators perform well in tracking the attentional state. Note that the sharp drop of the attention probability near $\sim 30\,\mathrm{s}$ in Row D is due to the fact that at each instance the real-time estimator does not observe the attention markers beyond the $1.5\,\mathrm{s}$ forward lag, whereas the batch-mode estimator estimates the probabilities given the entire trial. In case 2, the batch-mode estimator performs well even though the M100 components are not visually indicative of the attentional state. However, the classification confidence decreases considerably specially in the $(30, 60]$ s interval. The real-time estimator in case 2 closely follows the batch-mode estimator, but is more sensitive to the fluctuations of the extracted M100 components. Thus, its performance undergoes further degradation going from case 1 to 2, as compared with that of the batch-mode estimator. The red arrows in rows C and D of case 2 in Fig. S4 mark instances where the less robustness of real-time estimator resulted in misclassifications, while the batch-mode estimator classified the attended speaker correctly.

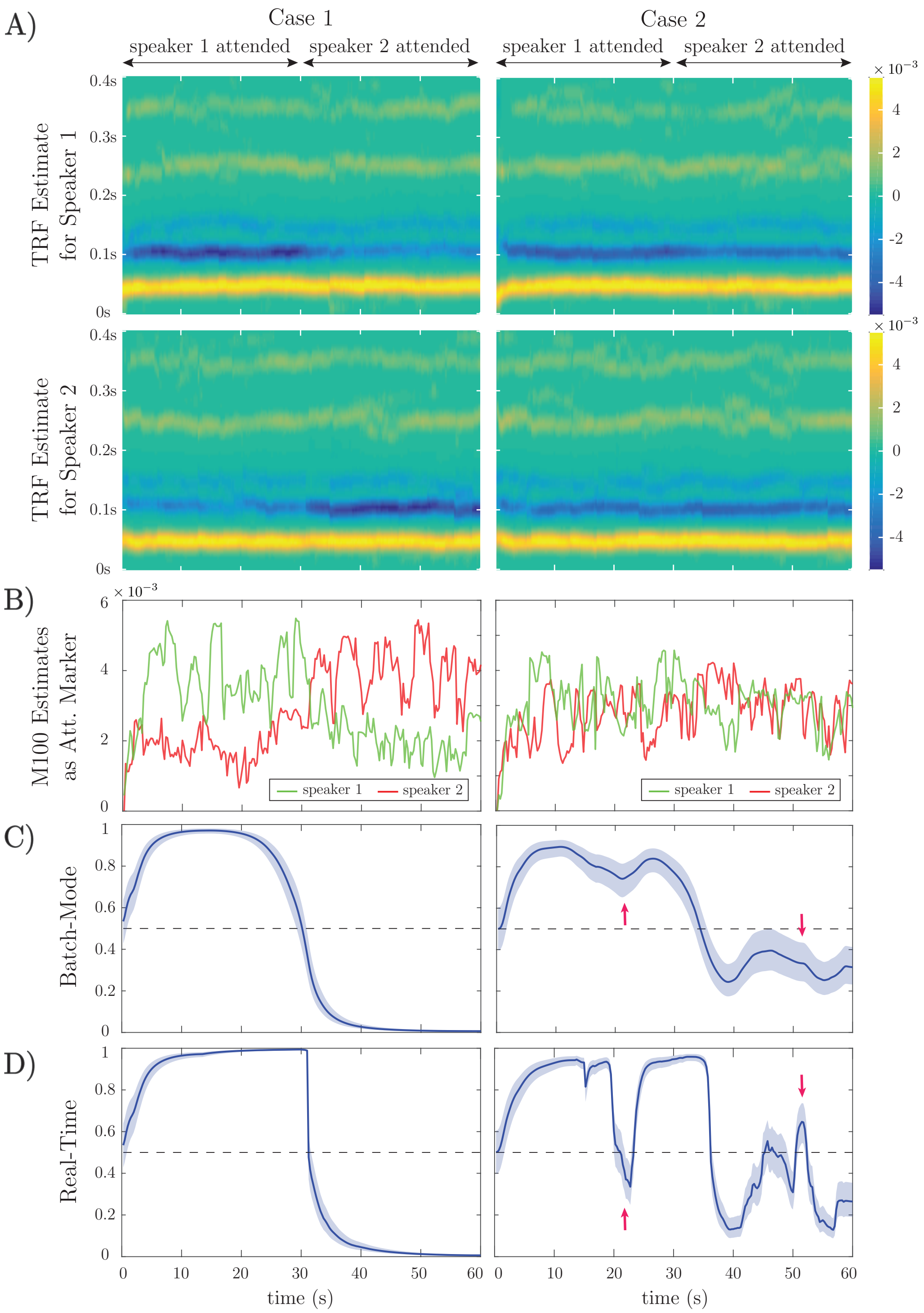

Figure S4. Estimation results of application to simulated MEG data: A) Estimated TRFs for case 1 (strong modulation in M100 components) and case 2 (weak modulation in M100 components). B) Estimated M100 magnitudes as the attention markers. C) Outputs of the batch-mode estimator as the estimated probability of attending to speaker 1. D) Outputs of the real-time estimator as the estimated probability of attending to speaker 1. The real-time estimator is less robust to the statistical fluctuations in the extracted M100 components, which can result in misclassifications as shown for two example instances marker by red arrows. However, it follows the general trend of the batch-mode estimator closely despite its online access to data.

It is worth noting that as we are using an encoding model in this case, the overall delay in estimating the attentional state is the forward-lag window, i.e., 1.5 s, and unlike the case of using the decoding model, the encoder lag does not contribute to the delay. Our analysis of the effect of K_F on the MSE of the real-time estimator with respect to the batch-mode was nearly identical to that presented for the EEG simulation, and is thus omitted for brevity.

REFERENCES

Akram, S., Presacco, A., Simon, J. Z., Shamma, S. A., and Babadi, B. (2016). Robust decoding of selective auditory attention from MEG in a competing-speaker environment via state-space modeling. *NeuroImage* 124, 906–917

Akram, S., Simon, J. Z., and Babadi, B. (2017). Dynamic estimation of the auditory temporal response function from MEG in competing-speaker environments. *IEEE Transactions on Biomedical Engineering* 64, 1896–1905

Babadi, B., Kalouptsidis, N., and Tarokh, V. (2010). SPARLS: The sparse RLS algorithm. *IEEE Transactions on Signal Processing* 58, 4013–4025

Combettes, P. L. and Pesquet, J.-C. (2011). Proximal splitting methods in signal processing. In *Fixed-Point Algorithms for Inverse Problems in Science and Engineering* (Springer New York). 185–212

De Jong, P. and Mackinnon, M. J. (1988). Covariances for smoothed estimates in state space models. *Biometrika* 75, 601–602

Ding, N. and Simon, J. Z. (2012a). Emergence of neural encoding of auditory objects while listening to competing speakers. *Proceedings of the National Academy of Sciences* 109, 11854–11859

Ding, N. and Simon, J. Z. (2012b). Neural coding of continuous speech in auditory cortex during monaural and dichotic listening. *Journal of Neurophysiology* 107, 78–89

Duchi, J. and Singer, Y. (2009). Efficient online and batch learning using forward backward splitting. *Journal of Machine Learning Research* 10, 2899–2934

Figueiredo, M. A., Nowak, R. D., and Wright, S. J. (2007). Gradient projection for sparse reconstruction: Application to compressed sensing and other inverse problems. *IEEE Journal of Selected Topics in Signal Processing* 1, 586–597

Goldstein, T., Studer, C., and Baraniuk, R. (2014). A field guide to forward-backward splitting with a FASTA implementation. *arXiv eprint* abs/1411.3406

Goldstein, T., Studer, C., and Baraniuk, R. (2015). FASTA: A generalized implementation of forward-backward splitting. `http://arxiv.org/abs/1501.04979`

Jenatton, R., Mairal, J., Bach, F. R., and Obozinski, G. R. (2010). Proximal methods for sparse hierarchical dictionary learning. In *Proceedings of the 27th International Conference on Machine Learning (ICML-10)*. 487–494

Sheikhattar, A., Fritz, J. B., Shamma, S. A., and Babadi, B. (2015). Recursive sparse point process regression with application to spectrotemporal receptive field plasticity analysis. *IEEE Transactions on Signal Processing* 64, 2026–2039

Smith, A. C. and Brown, E. N. (2003). Estimating a state-space model from point process observations. *Neural Computation* 15, 965–991

Smith, A. C., Frank, L. M., Wirth, S., Yanike, M., Hu, D., Kubota, Y., et al. (2004). Dynamic analysis of learning in behavioral experiments. *Journal of Neuroscience* 24, 447–461

Tanner, M. A. (1991). *Tools for Statistical Inference*, vol. 3 (Springer)
